# Desire to Die Communication Training for Professionals: Developing Online Formats

**DOI:** 10.1089/pmr.2024.0075

**Published:** 2025-04-10

**Authors:** Kathleen Boström, Thomas Dojan, Axel Doll, Thomas Montag, Raymond Voltz, Kerstin Kremeike

**Affiliations:** ^1^ Department of Palliative Medicine, Faculty of Medicine and University Hospital, University of Cologne, Cologne, Germany.; ^2^ Center for Integrated Oncology Aachen Bonn Cologne Duesseldorf (CIO ABCD), Faculty of Medicine and University Hospital, University of Cologne, Cologne, Germany.; ^3^ Center for Health Services Research, Faculty of Medicine and University Hospital, University of Cologne, Cologne, Germany.

**Keywords:** desire to die, online trainings, palliative and hospice care, website

## Abstract

**Background:**

Severely ill patients often express a desire to die, which can turn into suicidality. To support health professionals in managing this issue, we initially created a two-day face-to-face training to enhance self-confidence, knowledge, attitudes, and skills. Due to the increasing need for more accessible formats, we aimed to transition this training online and develop a complementary website.

**Methods:**

Multimethod approach to develop and evaluate an online training and a website on dealing with the desire to die in palliative care in Germany. This involved: (1) reviewing literature on effective didactic elements, (2) digitalizing the face-to-face training and creating the website with ongoing expert feedback, and (3) piloting and evaluating these resources through online surveys.

**Results:**

We retrieved suggestions for the development of online trainings and websites from *n* = 39 publications. Through these results and expert discussion, an online version of our training and a website were developed. For evaluation, we conducted two trainings (face-to-face (*n* = 8) and online (*n* = 19)) with multiprofessional participants. All improved significantly in self-confidence after the training without differences between online and face-to-face training. Website evaluation of usability, comprehension, information quality, presentation, and sustainability by *n* = 71 users yielded favorable feedback with improvement suggestions for structure and plain language.

**Conclusions:**

Dealing with the desire to die can be taught not only face to face but also through online training and an educational website. This can ensure low-threshold access to scientifically sound information and training units for those health professionals confronted with the desire to die.

## Background 

Patients with severe and life-limiting disease regularly report desires to die. As terminology and assessment vary, prevalence is hard to establish.^1^ A recent study found that 12% report such desires as temporary and 10% persistent.^2^ We apply a wide definition of desire to die along a continuum of increasing suicidal pressure to act. Our definition encompasses the acceptance of death, life satiety, and tiredness of life, as well as the (hypothetical) wish for hastened death and (in extreme forms) suicidality or the wish for (assisted) suicide.^3^ The quality and intensity of desires to die are prone to change over time and across situations.^4^ It may even be accompanied by a concurrent will to live.^3,5^ Desire to die is impacted by various physical, psychological, social, and spiritual factors such as depression, pain, or reduced quality of life.^1^

Health professionals in palliative and hospice care are often confronted with patients’ desires to die and report a rising need for support in dealing with the topic.^6^ To address this demand, we developed a multiprofessional training curriculum.^7^ Our two-day face-to-face training promotes theoretical knowledge, communicative skills, and self-reflection regarding desire to die. It also addresses the aforementioned impacting factors on the desire to die and respective interventions. The evaluation of the training yielded a stable increase of self-confidence in dealing with the desire to die even one year after participation.^8^ The training is still in high demand.

## Objective

Despite the high demand, attending a two-day face-to-face training is difficult to realize for many due to the high demands on personnel resources in palliative and hospice care.^9^ Additionally, legal regulations during the COVID-19 pandemic temporarily outlawed face-to-face events. To address these shortcomings, we pursued two objectives:

(1) Providing our trainings online to be more accessible for multiprofessional palliative care providers
(2) Developing a website for low-threshold self-study with engaging and informative content on dealing with desire to die

## Design

The online training and website to be developed are based on an existing two-day curriculum containing six units: 1. Exchange about Professional Practice, 2. Attitudes, Norms, and Values, 3. Scientific Perspective and Clinical Approach, 4. Reflection on Dealing with Desire to Die, 5. Dealing with Desire to Die—Practical Exercise, and 6. Self-Care.^7^ For digitalization and website development, we assessed content, didactic, and design elements of digital education formats for dealing with desire to die in palliative and hospice care. Our multimethod approach included:

(1) Literature search to gather relevant evidence of existing online formats
(2) Digitalization of the training and website development, accompanied by constant expert discussion within the project team
(3) Piloting and evaluation of the two developed (online) trainings and the website

### Literature search

In PubMed, we combined key terms related to *desire to die* and *online format*s. Identified publications underwent title–abstract screening and suitable publications were subsequently fed into an EndNote library for categorization of themes.^10^ The complete texts of categorized publications were examined in terms of *development*, *conduct*, and *evaluation* of digital education formats.

### Digitalization of the existing training and website development

Throughout the study, the project team supervised the implementation of results from the literature search for training digitalization and website development. This group consisted of *n* = 6 experts (course instructors of the existing training and research staff) representing expertise in medicine, nursing, psychology, social sciences, teaching, and training as well as palliative care. The project team discussed and consented all adaptations of the developed trainings. For the website development, expertise from the previous implementation of an online platform for research data regarding Health Services Research was incorporated as well.^11^

### Piloting and evaluation of the developed online training and website

To explore the effects of the digitalized desire to die training on participants, we planned two multiprofessional trainings: one face-to-face and one online, including the same content and comparable didactic methods. The corresponding website on dealing with desire to die was to be developed as part of the University Clinic of Cologne’s main website, to accommodate its regulations and to ensure sustainability.

### Measurements

For evaluation, a questionnaire on self-confidence in dealing with the desire to die was handed out before (t0) and right after (t1) the two trainings.^7^ The self-developed questionnaire contains 22 items on knowledge, skills, and attitudes regarding desire to die. For the online training, we transferred the questionnaire into an online survey using the platform LimeSurvey. Open questions at t1 were added to access participants’ views on the suitability of the online format.

For website evaluation, we also developed an online survey using the platform LimeSurvey. Based on results from the literature search on website development, we utilized items from existing questionnaires to cover the dimensions of interest, usability, comprehension, information quality, presentation, and sustainability.^12–14^ Ultimately, the online survey comprised 20 items on a 7-point Likert scale.^12^ Additionally, seven open questions allowed to gathering deeper information for further development. Except for question 7, all open questions were only triggered if participants rated the respective quantitative item 3 (“tend to disagree”) or less:

(1) What would you like to change about the existing website?
(2) Have you had any difficulties using the website?
(3) Why do you find the information on the website (rather) of poor quality?
(4) Why do you find the information on the website (rather) untrustworthy?
(5) What form of presentation would help you to better understand the content?
(6) Why would you not want to use the website in the future?
(7) Is there anything else we have not addressed that you would like to add?

Age, gender, belonging to the group of professional/volunteer/informal caregivers, as well as the electronic device used to access the website, were assessed as basic sociodemographic data.

## Results

The literature search and project team discussions yielded a pool of information that could be used for developing an online training format and a website. The implementation of results focused on applicability in the given context.

### Literature search

In July 2021, we identified *n* = 70 publications with our search strategy. After title-abstract screening, we included *n* = 38 for full-text screening, both by two authors (K.B. and T.D.). Full-text screening included another publication added through hand searching,^12^ as well publications on criteria as well as two quantitative scales for evaluation of educational websites.^13–18^ No existing online training on desire to die was identified. We summarized the results on *development* and *conducted* online education formats in Table 1.^19–50^

**Table 1.** Summary of Literature Search Results on Development and Conducting of Online Education Formats

Online trainings	
Technical tools	Didactic goals
Camera / Video conference	According to AVIVA-model^15^:
Chat tool and “reactions”	*A*rrive
“Breakout rooms”	*Asses prior knowledge*
Mobile phones	*Inform*
	*Apply*
	*Analyze*

Educational websites	
Teaching methods	Available media
Case vignettes	Text
Role-play	Picture
Expert opinions and discussions	Video and audio
Information, Publications	Interactive platforms
Presentations, talks	
communication guidelines	
Formulation aids	

*Evaluation* criteria exist for: (1) user satisfaction (efficiency, attractiveness), (2) quality of video material, (3) quality of online material, as well as (4) usage time.^13,14^ Based on these and with the inclusion of items from the *Federal Center for Health Education* (BZgA) toolbox identified by handsearching,^12^ we developed a questionnaire to evaluate our educational website (see Appendix Table A1).

### Digitalization of the existing training program

The project team held three meetings to elaborate a strategy for digitizing the curriculum of the two-day training utilizing the results of the literature research. Subsequently, a concept for (online) training on dealing with the desire to die and a corresponding website was developed.

**FIG. 1.**
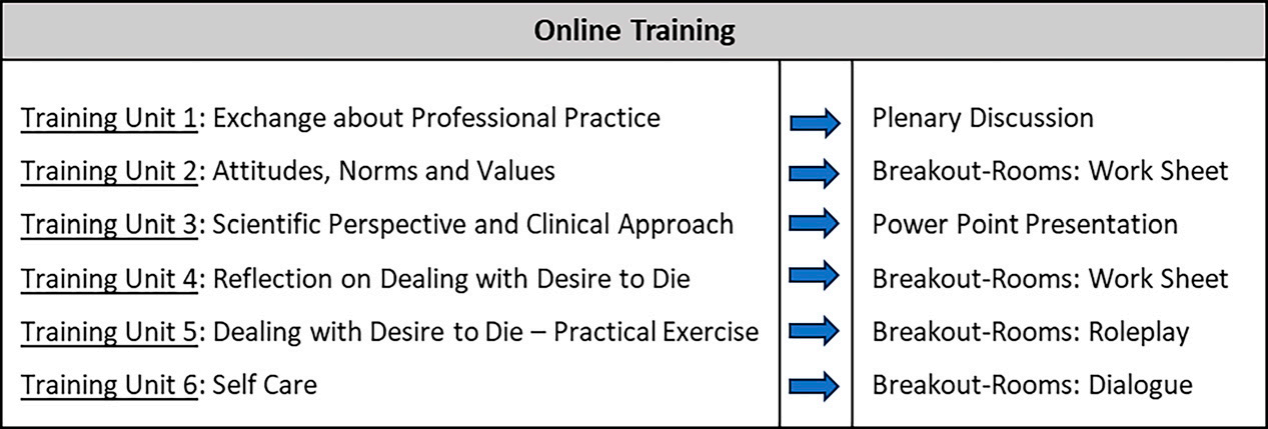
Structure and used didactic approach of the online training, based on the original six training units1.

The current course curriculum was adapted for an online conduction. Using the communication platform Zoom,^51^ all six original education units could be digitized. For structure and the used didactic approaches of the online training, please see Figure 1.

Results from the literature search that were implemented include recommendations concerning technical tools (video conference, chat tool and reactions, “breakout rooms”) and teaching methods (case vignettes, role-play information, publications, and presentations). This multimedia and multimethod approach was selected because it most closely resembles the interaction possibilities of our original training. Results from Table 1 that were rejected by the project team due to impracticability (e.g., due to financial and timely limitations) include didactic goals of the *AVIVA*-rationale,^15^ inclusion of analog media, networking via other channels, and networking for participants.

### Development of a low-threshold website

To provide low-threshold access to the desire to die knowledge, an educational website was created (accessible via *palliativzentrum.uk-koeln.de/umgang-mit-todeswuenschen*). A self-study area provides information about desire to die, and there are information and registration options for various training formats.

**FIG. 2.**
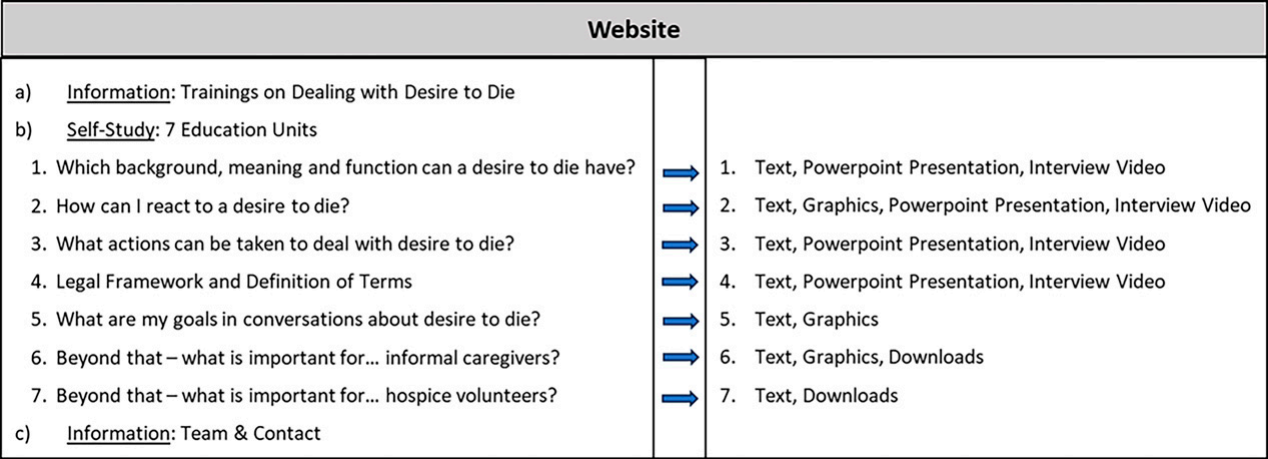
Structure and used media of the developed website, based on the original six training units.

Besides information on all six content units of the original training curriculum presented for self-study, two new topics were added: a self-study unit for informal caregivers provides material and information as well as contact addresses of hospice associations and self-help groups. Another self-study unit explores the role and special availabilities of hospice volunteers. These topics were added due to the special demand by participants of previous trainings. For structure and used media of the final website, please see Figure 2.

Implemented results from the literature search include recommendations on media: all units used text and videos or informational graphics, such as interviews with experts from the research team and slides with voice-over. The project team finalized the website’s layout and language through multiple rounds of internal feedback. To guarantee lay comprehension, an external media communication agency also revised the website texts. These technological tools were used to counter text heaviness and promote engagement with the website.

#### Piloting and evaluation of developed online training and website

##### Evaluation of the online training

To evaluate the developed online desire to die training, two training courses were held for multiprofessional palliative care providers: a two-day face-to-face training with *n* = 8 participants employed at a local interdisciplinary palliative care center and a structurally identical two-day online training with *n* = 19 multiprofessional participants working for an ambulatory palliative and hospice service in home palliative care. Table 2 shows sociodemographic information of participants of the two conducted trainings.

**Table 2.** Sociodemographic Data for All Participants of the (Online) Multiprofessional Trainings

	Multiprofessional	
	Face-to-face	Online
Age		
<=39	1	6
40–49	2	2
50–59	4	8
>=60	1	3
Not specified	/	1
Gender		
Female	7	15
Male	1	4
Not specified	/	1
Profession^a^		
Physician	1	3
Nursing	0	8
Psychosocial	3	3
Volunteer	2	1
Informal caregiver^b^	2	4
Other	/	4

^a^ Multiple answers possible.

^b^ All participants who chose the “informal caregiver” option also chose another profession as a primary option.

Participants of the face-to-face and online trainings completed the evaluation questionnaire before and directly after the training.^7^ As mean values were not normally distributed (Shapiro–Wilk test up to *p* = <.001), we carried out a Wilcoxon test to compare mean values. Mean values and standard deviations of all items can be found in Table 3.

**Table 3.** Mean Values and Standard Deviations (in Brackets) of All Items at t0 (Before Training) and t1 (After Training) for the Face-to-Face and Online Trainings of Multiprofessional Palliative Care Providers

Nr	Item	Face-to-face (*n* = 8)			Online (*n* = 20)		
		t0	t1	*p* Value	t0	t1	*p* Value
1	How confident do you feel to discuss a patient’s desire to die?	4.29 (1.98)	**5.83** ^a^ **(1.47)**	0.031	4.18 (1.33)	**5.56** ^a^ **(0.88)**	0.031
2	I would feel discomfort in discussing desire to die with patients.	2.00 (1.19)	1.63 (0.74)	0.142	2.35 (1.06)	**1.78** ^a^ **(0.83)**	0.025
3	Desire to die discussions are not possible for me due to lack of time.	1.50 (0.93)	1.38 (0.52)	0.366	. .	. .	. .
4	I am afraid that a discussion with a patient about his/her desire to die would affect me too deeply.	1.25 (0.46)	1.25 (0.46)	0.500	2.29 (1.26)	1.67 (1.32)	0.215
5	I am able to address desire to die proactively with a patient.	2.75 (0.46)	**1.75** ^a^ **(0.71)**	0.004	2.41 (1.00)	2.41 (1.00)	0.364
6	I know different ways of reacting to patients with a desire to die.	3.00 (0.93)	**3.87** ^a^ **(1.25)**	0.010	3.00 (1.09)	**4.11** ^a^ **(0.93)**	0.031
7	I am able to use different approaches to respond to patients with desires to die.	3.25 (0.89)	3.37 (1.30)	0.381	3.00 (1.03)	**4.33** ^a^ **(0.71)**	0.008
8	I know several possible backgrounds to a desire to die.	3.75 (0.89)	3.75 (1.49)	0.342	3.94 (1.03)	4.56 (1.01)	0.096
9	I know several possible functions of a desire to die.	3.38 (0.92)	3.62 (1.68)	0.164	3.37 (1.26)	4.33 (1.00)	.085
10	I am familiar with the current legal situation regarding physician-assisted suicide, allowing people to die and euthanasia.	3.75 (1.03)	3.75 (1.49)	0.070	3.94 (1.14)	4.33 (1.32)	0.136
11	I am unsure about my duty of care with suicidal patients.	2.25 (1.03)	2.88 (1.36)	0.201	2.75 (1.18)	2.33 (0.71)	0.500
12	I know the key points of relevant recommendations for dealing with desires to die.	2.88 (1.13)	3.75 (1.28)	0.299	3.06 (1.53)	4.22 (1.09)	0.297
13	I know signs that indicate acute suicidal tendencies in patients.	3.00 (1.19)	**3.63** ^a^ **(0.91)**	0.017	3.37 (1.02)	**4.00** ^a^ **(0.71)**	0.012
14	I recognize signs of own exhaustion when confronted with desire to die.	3.50 (1.07)	3.88 (0.99)	0.500	3.35 (1.06)	3.44 (1.24)	0.112
15	I can manage to not exhaust myself when patients express a desire to die.	3.62 (0.92)	3.75 (1.28)	0.500	3.50 (1.16)	3.67 (0.87)	0.219
16	I am not aware of my own attitude to the subject of desire to die.	1,88 (0.83)	2.63 (1.99)	0.077	2.06 (1.44)	1.78 (1.39)	0.059
17	I am aware of my fears when dealing with patients with desire to die.	3.62 (0.74)	4.13 (0.64)	0.189	3.63 (1.02)	4.44 (0.73)	0.279
18	When a patient asks me for help in dying I discuss this desire to die with the patient in detail.	4.00 (0.93)	4.38 (0.74)	0.175	3.71 (1.31)	**4.78** ^a^ **(0.44)**	**0.048**
19	When I am confronted with a desire to die, I feel helpless.	2.13 (0.64)	1.75 (0.71)	0.142	2.00 (1.06)	**1.22** ^a^ **(0.44)**	**0.**009
20	When I am confronted with a desire to die, I want to flee the situation.	1.50 (0.53)	1.50 (0.76)	0.500	1.82 (1.13)	1.78 (1.39)	0.424
21	I am able to accept patients with their desires to die.	4.00 (0.93)	4.13 (1.46)	0.408	3.69 (1.08)	4.33 (1.00)	0.173
22	I am able to stay in contact with patients with desires to die.	4.00 (1.07)	4.13 (1.46)	0.431	3.63 (1.15)	4.22 (0.97)	0.199

^a^ Significant with *p* ≤ 0.05.

Bold data is significant with *p* ≤ 0.05.

Multiprofessional palliative care providers showed significant improvement between t0 and t1 after both trainings, in 4 and 6 of 22 items, respectively. In this small sample size, we exploratorily tested for differences and found no significant differences at t0 and t1 between the face-to-face and the online training group.

Participants of the online training were asked to answer open questions regarding the suitability of the digital format. Answers were predominantly positive, emphasizing the “*broader access*”. Regarding content, participants reported ambivalent requests regarding information on the topic of assisted suicide (“*Go into more detail about assisted suicide and give some examples.*” and “*A little less assisted suicide, more ‘simple’ desires to die*”).

### Website evaluation

The website link was sent to mailing lists of stakeholders already known to the research team with a request for evaluation. With two reminders, overall, 244 interested parties could be contacted (individuals and mailing lists). They were asked to share the website and the invitation to evaluate it via their networks.

A total of *n* = 71 participants completed the online survey consisting of open and closed questions. The vast majority visited the website via their PC (*n* = 56; 78.9%), few used a tablet (*n* = 4; 5.6%), or smartphone (*n* = 8; 11.3%). Participants were mostly female (*n* = 55, 55.5%), ages varied between 18–39 years (*n* = 10, 15,4%), 40–59 years (*n* = 33, 50.7%), and 60 to over 70 years (*n* = 22, 33.9%). Most participants reported to work as a professional in palliative and hospice care (*n* = 55, 79.5%), with only a few being volunteers (*n* = 14, 20.3%). No one reported to be an informal caregiver.

In a range from 1 “*very good*” to 5 “*inadequate*,” users rated the website with an overall mean grade of 2.24 (SD = 1.2). For mean ratings on all items of the five dimensions usability, comprehension, information quality, presentation, and sustainability on a Likert-scale from 1 “*completely disagree*” to 7 “*completely agree*,” please see Table 4.

**Table 4.** Mean Ratings with Standard Deviations of All Items from the Online Survey on Website Evaluation

Nr	Item	M (SDs)
Interest		
1	I find the content of this website interesting.	6.23 (1.03)
Usability		
2	I think this website is easy to use.	6.04 (1.03)
3	I think this website is well designed.	5.83 (1.23)
4	I find the website easy to understand.	6.22 (1.07)
5	It is easy for me to find the information I amlooking for.	5.93 (1.20)
6	I can quickly access the information I need.	6.03 (1.12)
Comprehension		
8	The individual sentences are easy to read.	6.07 (1.14)
9	The texts provide me with the most important information briefly and concisely.	6.09 (1.04)
10	The language used in the texts is familiar and generally understandable.	5.94 (1.13)
Information Quality		
11	The information on the website is of high quality.	6.10 (1.11)
12	The information on the website is trustworthy.	6.25 (1.08)
13	I find the information on the website useful for my day-to-day work.	5.74 (1.27)
Presentation		
14	The website prepares research results well for practical use.	5.73 (1.13)
15	I find the presentation formats (e.g., illustrations, tables, and videos) helpful for understanding the content.	5.70 (1.34)
Sustainability		
16	I will use this website again.	5.93 (1.23)
17	I will visit this website regularly.	4.83 (1.62)
18	I would recommend this website to colleagues, friends, and acquaintances.	5.92 (1.25)
19	If I am interested in such topics in the future, I could imagine visiting this website again.	6.14 (1.07)

M, mean; SDs, standard deviations.

A few participants (*n* = 13) used the possibility to give feedback on the open-ended questions. Repeated requests concerned an even more visibly structured layout [“*Use subheadings (especially on the introductory page)*”] as well as an easier language for laypeople (“*I wish for paragraphs or the whole website written in easy language”*). A few users expressed confusion upon the connection to and corporate design of the official website of the University of Cologne, claiming that “*it is not logical or helpful*”. All in all, most open feedback emphasized the value of the data provided and gave suggestions for further improvement, e.g., by adding further topics such as the desire to die in old age or in children and youth.

## Conclusions

International legal trends increasingly support a liberal approach to assisted suicide,^52^ making it essential for health professionals to effectively address patients’ end-of-life desires. Following a two-day training we developed, health professionals demonstrated enhanced self-confidence, knowledge, skills, and attitudes.^7^ Despite a substantial demand for both in-person and online training (we conducted 35 trainings between 2021 and 2024), the workload constraints of staff have limited their ability to attend face-to-face sessions.^53^ Consequently, we aimed to address this demand, informed by our evaluated face-to-face curriculum.^7,8^ Using literature search and expert discussions, we were able to develop an online version of the training and a low-threshold educational website.

Exploratory evaluation of our digital training indicates no significant difference in outcomes between online and face-to-face formats, consistent with other end-of-life trainings.^54^ Online trainings offer advantages, such as reduced costs, flexibility for participants, and effective use of technology, for in-depth discussions of existential topics. However, feedback highlighted a lack of informal team-building opportunities, such as coffee breaks. The provision of online trainings can therefore be seen rather as one additional possibility among education offers. While online training expands educational options, some participants sought more detailed guidance on handling requests for assisted dying, both inpatient interactions and institutional ethics. As requests for assisted dying increase, this need becomes urgent.^55^ We view the desire to die as a broader phenomenon, with realized assistance in dying as an extreme and rare expression.^3^ However, health professionals in many countries are confronted with requests for assisted dying.^56^ Therefore, understanding and dealing with the desire to die should be fundamental—independent of national legal regulations. Further development of trainings for institutions to find an ethical and practical stance maybe helpful.

To provide optimal care for patients expressing a desire to die, it is essential to offer easily accessible online information. Educational websites are increasingly used to convey medical and health information to both professionals and the public.^57^ While social media is popular for health information, it often suffers from low quality and misinformation.^58,59^ Our website offers free, low-threshold, evidence-based information on dealing with desire to die and has been rated “good” on the main criteria of website quality (usability, comprehension, information quality, presentation, and sustainability).^12–14^ The credibility of our website is enhanced by professional credentials and reliable sources.^60^ In a landscape where health information online can be confusing and misleading, source credibility can be an important demarcation from low-quality information websites.^61^ This is particularly valuable in the emotionally charged discourse on medical assistance in dying.

Website ratings might be impacted by selection bias, as our evaluation sample was already known to the research team. However, participants also gave a substantial amount of criticism: user evaluations suggest that, despite using videos, graphics, and text, our website prioritizes “essence over esthetics”.^62^ Health professionals in our evaluation and other studies highlight the need for more diverse media use to enhance accessibility.^63^ Additionally, feedback often points to the need for simpler, more inclusive language, as plain language has been shown to improve accessibility.^64^ To accommodate this need, future development should include plain language text alternatives and extensive pretesting, beyond the efforts of a media agency focused on accessible health information. Connecting the website to the server and design format of the University Clinic Cologne, we faced the challenge of limited possibilities for creative design. However, it also enables the research team to keep website information up-to-date according to the current legal framework and best-practice advice, independent on individual project funding.

## Strengths and Limitations

The major strength of our study lies within the sustainability of the developed trainings on dealing with the desire to die through its possibility for continuous further development according to current needs. By drawing on an already existing successful training curriculum for adaptation, we tried to ensure that existing knowledge becomes available to a larger audience. For website development, we could draw on a previous project on the translation of research data for health care practice,^11^ thereby making use of experiences on website development.

Our study is limited in several ways: With a literature search in PubMed (Medline) only, we might have overlooked relevant publications in other databases.^65^ The questionnaire used for online training evaluation is not validated. Also, contrary to our face-to-face-trainings,^8^ we cannot be sure of the practice transfer learned knowledge and skills from our online trainings yet. Additionally, our sample size is small and limited in its generalizability (e.g., age and gender distribution). While developing the website, we were limited by the necessary connection to the website domain of our employing institution. Although the use of its corporate design likely increased credibility, it also limited possibilities in multimedia use.

As a perspective for future research, it is important to ensure adequate education in dealing with the desire to die for an even wider audience. Therefore, effects of the developed training should be evaluated using both a larger and more diverse sample size. This could also adequately assess the effectiveness of online trainings compared to face-to-face trainings. To also reach target groups like hospice volunteers or informal caregivers of people with severe and life-limiting illnesses would require a tailored approach specific in content, form, and language^560^. This issue is especially true for elderly persons or those with a language barrier.^66^

## Ethics Approval and Consent to Participate

All research was conducted according to the Declaration of Helsinki and received a favorable vote from the ethics committee of the University of Cologne (Nr. 21-1412_1, 04.11.2022). All participants gave written informed consent.

## Abbreviations Used

AVIVA: Arrive and tune in, activate prior knowledge, inform, process, evaluate
BZgA: Federal Center for Health Education
CIO ABCD: Center for Integrated Oncology Aachen Bonn Cologne Duesseldorf
G-BA: Federal Joint Committee

## Appendix 1

### Questionnaire used for Website Evaluation

**Table A1.** All questions had the same response options as question 1, except for questions 10, 16, 18, 20, 23, 28, 29 and 30. Question 29 used response options based on the German school rating system, the other questions were open questions

Nr	Questions
1	I find the content of this website interesting.
	• No answer possible • Completely disagree • Disagree • Slightly disagree • Neutral • Slightly agree • Agree • Completely Agree
2	I think this website is easy to use.
3	I think this website is easy to use.
4	I think this website is easy to use.
5	I think this website is attractively designed.
6	I find using the website intuitive.
7	It is easy for me to find the information I am looking for.
8	I can access the information I am looking for quickly.
9	Would you want to change anything about the structure or functions of the website for regular use?
10	Open Question: What would you like to change about the existing website?
11	Have you had any difficulties using the website?
12	The individual sentences are easy to read.
13	The texts provide me with the most important information briefly and concisely.
14	The language used in the texts is familiar and generally understandable.
15	The information on the website is of high quality.
16	Open Question: Why do you find the information on the website (rather) poor quality?
17	The information on the website is trustworthy.
18	Open Question: Why do you find the information on the website (rather) untrustworthy?
19	I find the information on the website useful for my everyday work.
20	Open Question: What information would you like to have for your everyday work (practical relevance)?
21	The website presents research results well for practical use.
22	I find the presentation formats helpful for understanding the content.
23	Open Question: Which presentation formats would help you to better understand the content?
24	I will use this site again.
25	I will visit this site regularly.
26	I would recommend this website to colleagues, friends and acquaintances.
27	If I am interested in these topics in the future, I could imagine visiting the website again.
28	Open Question: Why would you not want to use this website in the future?
29	All in all: I give this website an overall rating of...
	1 - very good 2 – good 3 - satisfactory 4 - sufficient 5 – inadequate 6 - unsatisfactory
30	Open Question: Is there anything else we haven't mentioned that you would like to add?

Sociodemographics
How old are you?
• 18-29 • 30-39 • 40-49 • 50-59 • 60-69 • ≥70
You are…
• Female • Male • Diverse
Which user group would you consider yourself to be in?
• Professional • Volunteers • Relative
What device did you use to visit the website?
• Computer / Laptop • Tablet • Smartphone

## References

[1] Rodríguez-Prat A, Pergolizzi D, Crespo I, et al. The wish to hasten death in patients with life-limiting conditions. A systematic overview. Journal of Pain and Symptom Management. 2024;68(2):e91–e115. 38703862 10.1016/j.jpainsymman.2024.04.023

[2] Wilson KG, Dalgleish TL, Chochinov HM, et al. Mental disorders and the desire for death in patients receiving palliative care for cancer. BMJ Support Palliat Care 2016;6(2):170–177. 10.1136/bmjspcare-2013-000604 24644212

[3] German Guideline Program in Oncology (GGPO). Extended S3 Guideline Palliative care for patients with incurable cancer, Short version 2.2 – September 2020; AWMF-Registration number: 128/001OL; Available from: https://www.leitlinienprogramm-onkologie.de/fileadmin/user_upload/Downloads/Leitlinien/Palliativmedizin/Version_2/GGPO_Palliative_Care_ShortVersion_2.2.pdf

[4] Voltz R, Galushko M, Walisko J, et al. Issues of “life” and “death” for patients receiving palliative care-comments when confronted with a research tool. Support Care Cancer 2011;19(6):771–777. 20422231 10.1007/s00520-010-0876-z

[5] Boström K, Dojan T, Hellmich M, et al. The double awareness of the wish to hasten death and the will to live: A secondary analysis of outlier patients from a mixed-methods study. Palliat Med 2024;38(9):1042–1053. 39152645 10.1177/02692163241269689 PMC11487875

[6] Galushko M, Frerich G, Perrar KM, et al. Desire for hastened death: How do professionals in specialized palliative care react? Psychooncology 2016;25(5):536–543. 26374399 10.1002/pon.3959

[7] Frerich G, Romotzky V, Galushko M, et al. Communication about the desire to die: Development and evaluation of a first needs-oriented training concept—A pilot study. Palliat Support Care 2020;18(5):528–536. 32131932 10.1017/S1478951520000097

[8] Boström K, Dojan T, Frerich G, et al. Umgang mit Todeswünschen in der Palliativversorgung—Evaluation eines Schulungsprogramms. Zeitschrift Für Palliativmedizin 2022;23(04):198–206.

[9] Blätzinger M. Digitale weiterbildung—Herausforderung in der aktuellen situation. OP-Journal 2022;38(01):24–28.

[10] The EndNote Team. EndNote. EndNote. 20 ed. Philadelphia, PA: Clarivate; 2013.

[11] PMV Forschungsgruppe. CoRe-Web. Mit Daten und Fakten für eine bessere Gesundheitsversorgung in Köln. Available from: https://coreweb.pmvforschungsgruppe.de/ [Last accessed: 29 July, 2024].

[12] Thielsch MT. (with work from Salaschek M. Toolbox zur kontinuierlichen Website-Evaluation und Qualitätssicherung (Version 2.1). Arbeitsbericht, Köln: Bundeszentrale für gesundheitliche Aufklärung (BZgA). 2017.

[13] Lewis J, Utesch B, Maher D. UMUX-LITE: When there’s no time for the SUS2013 pp. 2099–2102.

[14] Venkatesh V, Bala H. Technology acceptance model 3 and a research agenda on interventions. Decision Sciences 2008;39(2):273–315.

[15] Städeli C, Grassi A, Rhiner K, Obrist W. Kompetenzorientiert unterrichten. Das AVIVA-Modell. Bern: hep; 2010.

[16] Chan TM, Grock A, Paddock M, et al. Examining Reliability and Validity of an Online Score (ALiEM AIR) for Rating Free Open Access Medical Education Resources. Ann Emerg Med 2016;68(6):729–735. 27033141 10.1016/j.annemergmed.2016.02.018

[17] Koch LF, Faßhauer U, Reiber K. [E-Learning in bachelor-level nursing education in Germany and the role of the nurse educator—a Delphi survey]. Pflege 2019;32(1):31–46. 30523757 10.1024/1012-5302/a000653

[18] Van Nuland SE, Eagleson R, Rogers KA. Educational software usability: Artifact or Design? Anat Sci Educ 2017;10(2):190–199. 27472554 10.1002/ase.1636

[19] Archambault PM. WikiBuild: A new application to support patient and health care professional involvement in the development of patient support tools. J Med Internet Res 2011;13(4):e114. 22155746 10.2196/jmir.1961 PMC3278100

[20] Castillo S, Calvitti K, Shoup J, et al. Production Processes for Creating Educational Videos. CBE Life Sci Educ 2021;20(2):es7. 33944619 10.1187/cbe.20-06-0120 PMC8734383

[21] Latha K, Meena KS, Pravitha MR, et al. Effective use of social media platforms for promotion of mental health awareness. J Educ Health Promot 2020;9:124. 32642480 10.4103/jehp.jehp_90_20 PMC7325786

[22] Lauruska V, Kubilinskas E. A system for teleconsulting, communication and distance learning for people with disabilities. J Telemed Telecare 2002;8(Suppl 2):49–50. 12217133 10.1177/1357633X020080S222

[23] McConville SA, Lane AM. Using on-line video clips to enhance self-efficacy toward dealing with difficult situations among nursing students. Nurse Educ Today 2006;26(3):200–208. 16300862 10.1016/j.nedt.2005.09.024

[24] Turton BM, Williams S, Burton CR, Williams L. Arts-based palliative care training, education and staff development: A scoping review. Palliat Med 2018;32(2):559–570. 28604224 10.1177/0269216317712189

[25] Youngblood P, Harter PM, Srivastava S, et al. Design, development, and evaluation of an online virtual emergency department for training trauma teams. Simul Healthc 2008;3(3):146–153. 19088658 10.1097/SIH.0b013e31817bedf7

[26] Lautenschlager NT, Diehl-Schmid J, Loi SM, et al. Modern technology to support carers of care recipients with dementia or functional mental illness: Promising progress, but a long road ahead. Int Psychogeriatr 2017;29(12):1933–1935. 29130870 10.1017/S1041610217002150

[27] Shin JY, Choi SW. Online interventions geared toward increasing resilience and reducing distress in family caregivers. Curr Opin Support Palliat Care 2020;14(1):60–66. 31842019 10.1097/SPC.0000000000000481 PMC6996606

[28] Wittenberg E, Alabere RO, Beltran E, et al. Sharing COMFORT Communication Training With Healthcare Professionals in Nairobi, Kenya: A Pilot Webinar Series. The American Journal of Hospice & Palliative Care 2021;10499091211026673. 10.1177/10499091211026673 34159800

[29] Wittenberg E, Goldsmith JV, Williams Y, Lee A. Caring for Family Caregivers: A Pilot Test of an Online COMFORT™ (SM) Communication Training Module for Undergraduate Nursing Students. J Cancer Educ 2020;35(1):138–143. 30467775 10.1007/s13187-018-1452-3 PMC6533166

[30] Arenella C, Yox S, Eckstein DS, Ousley A. Expanding the reach of a cancer palliative care curriculum through Web-based dissemination: A public-private collaboration. J Cancer Educ 2010;25(3):418–421. 20237885 10.1007/s13187-010-0066-1

[31] Barton MB, Thode RJ. Distance learning in the Applied Sciences of Oncology. Radiother Oncol 2010;95(1):129–132. 20223541 10.1016/j.radonc.2010.02.011

[32] Bishop CT, Mazanec P, Bullington J, et al. Online End-of-Life Nursing Education Consortium Core Curriculum for Staff Nurses: An Education Strategy to Improve Clinical Practice. J Hosp Palliat Nurs 2019;21(6):531–539. 31568109 10.1097/NJH.0000000000000593

[33] Bramstedt KA, Prang M, Dave S, et al. Telemedicine as an ethics teaching tool for medical students within the nephrology curriculum. Prog Transplant 2014;24(3):294–297. 25193732 10.7182/pit2014289

[34] Chappell PM, Healy J, Lee S, et al. Communicating With Dying Patients and Their Families: Multimedia Training in End-of-Life Care. Am J Hosp Palliat Care 2017;34(7):637–644. 27384610 10.1177/1049909116655293

[35] Clabburn O, Groves KE, Jack B. Virtual learning environment (‘Ivy Street’) for palliative medicine education: Student and facilitator evaluation. BMJ Support Palliat Care 2020;10(3):318–323. 10.1136/bmjspcare-2019-002159 32461222

[36] Donovan AK, Wood GJ, Rubio DM, et al. Faculty Communication Knowledge, Attitudes, and Skills Around Chronic Non-Malignant Pain Improve with Online Training. Pain Med 2016;17(11):1985–1992. 27036413 10.1093/pm/pnw029

[37] Fleetwood J, Vaught W, Feldman D, et al. MedEthEx Online: A computer-based learning program in medical ethics and communication skills. Teach Learn Med 2000;12(2):96–104. 11228685 10.1207/S15328015TLM1202_7

[38] Juraskova I, Laidsaar-Powell R, Keast R, et al. eTRIO trial: Study protocol of a randomised controlled trial of online education modules to facilitate effective family caregiver involvement in oncology. BMJ Open 2021;11(5):e043224. 10.1136/bmjopen-2020-043224 PMC8166623 34049902

[39] Loerzel VW, Conner N. Advances and Challenges: Student Reflections From an Online Death and Dying Course. Am J Hosp Palliat Care 2016;33(1):8–15. 25172782 10.1177/1049909114549182

[40] Lunsford B, Posey L. Geriatric education utilizing a palliative care framework. Gerontol Geriatr Educ 2018;39(2):183–192. 28129090 10.1080/02701960.2017.1285293

[41] Macqueen S, Woodward-Kron R, Flynn E, et al. A resource for teaching emergency care communication. Clin Teach 2016;13(3):192–196. 26183768 10.1111/tct.12423

[42] Pasacreta JV, Kenefick AL, McCorkle R. Managing distress in oncology patients: Description of an innovative online educational program for nurses. Cancer Nurs 2008;31(6):485–490. 18987517 10.1097/01.NCC.0000339251.83571.f9

[43] Pelayo M, Cebrián D, Areosa A, et al. Effects of online palliative care training on knowledge, attitude and satisfaction of primary care physicians. BMC Fam Pract 2011;12:37. 21605381 10.1186/1471-2296-12-37 PMC3123578

[44] Rawlings D, Winsall M, Yin H, Devery K. What is a compassionate response in the emergency department? Learner evaluation of an End-of-Life Essentials online education module. Emerg Med Australas 2021. 10.1111/1742-6723.13776 PMC9292911 33951282

[45] Rawlings D, Winsall M, Yin H, et al. Evaluation of an End-of-Life Essentials Online Education Module on Chronic Complex Illness End-of-Life Care. Healthcare (Basel) 2020;8(3). 10.3390/healthcare8030297 PMC7551176 32854394

[46] Robertson AC, Fowler LC, Niconchuk J, et al. Application of Kern’s 6-Step Approach in the Development of a Novel Anesthesiology Curriculum for Perioperative Code Status and Goals of Care Discussions. J Educ Perioper Med 2019;21(1):E634. PMC6685461 31406705

[47] Rogers MM, Chambers B, Esch A, et al. Use of an Online Palliative Care Clinical Curriculum to Train U.S. Hospital Staff: 2015–2019. J Palliat Med 2021;24(4):488–495. 33306934 10.1089/jpm.2020.0514

[48] Schmitz CC, Braman JP, Turner N, et al. Learning by (video) example: A randomized study of communication skills training for end-of-life and error disclosure family care conferences. Am J Surg 2016;212(5):996–1004. 27474496 10.1016/j.amjsurg.2016.02.023

[49] Wheeler C, Anstey S, Lewis M, et al. The effect of education on community nursing practice in improving the patient-carer experience at the end of life. Br J Community Nurs 2014;19(6):284–286. 24902056 10.12968/bjcn.2014.19.6.284

[50] Wittenberg-Lyles E, Goldsmith J, Ferrell B, Burchett M. Assessment of an interprofessional online curriculum for palliative care communication training. J Palliat Med 2014;17(4):400–406. 24401084 10.1089/jpm.2013.0270

[51] Zoom Video Communications. Zoom Meetings. 2011.

[52] Buchbinder M, Cain C. Medical Aid in Dying: New Frontiers in Medicine, Law, and Culture. Annu Rev Law Soc Sci 2023;19(1):195–214.

[53] Müller H, Kiepke-Ziemes S, Münch U. Jetzt handeln: Personal im Gesundheitswesen vor Burnout!. Zeitschrift Für Palliativmedizin 2021;22(03):129–130.

[54] Day FC, Srinivasan M, Der-Martirosian C, et al. A Comparison of Web-Based and Small-Group Palliative and End-of-Life Care Curricula: A Quasi-Randomized Controlled Study at One Institution. Academic Medicine 2015;90(3):331–337. 25539518 10.1097/ACM.0000000000000607 PMC4340770

[55] Batzler YN, Melching H, Schallenburger M, et al. Beweggründe für den Wunsch nach Suizidassistenz. Eine retrospektive Auswertung telefonischer Anfragen. (Reasons for wanting assisted suicide—a retrospective evaluation of telephone inquiries). Deutsches Arzteblatt International 2023;120:754–755. 38014440 10.3238/arztebl.m2023.0178 PMC10722489

[56] Schildmann J, Cinci M, Kupsch L, et al. Evaluating requests for physician-assisted suicide. A survey among German oncologists. Cancer Med 2023;12(2):1813–1820. 35770954 10.1002/cam4.4981 PMC9883542

[57] Monkman H, Schmidt T, Nøhr C. Online Medication Information for Citizens: A Comparison of Demands on eHealth Literacy. Stud Health Technol Inform 2020;270:1026–1030. 32570537 10.3233/SHTI200317

[58] Afful-Dadzie E, Afful-Dadzie A, Egala SB. Social media in health communication: A literature review of information quality. Health Inf Manag 2023;52(1):3–17. 33818176 10.1177/1833358321992683

[59] McMahon KM, Schwartz J, Nilles-Melchert T, et al. YouTube and the Achilles Tendon: An Analysis of Internet Information Reliability and Content Quality. Cureus 2022;14(4):e23984. 35573564 10.7759/cureus.23984 PMC9091342

[60] Wollmann K, der Keylen PV, Tomandl J, et al. The information needs of internet users and their requirements for online health information—A scoping review of qualitative and quantitative studies. Patient Educ Couns 2021;104(8):1904–1932. 33563502 10.1016/j.pec.2021.01.020

[61] Daraz L, Bouseh S. Developing a quality benchmark for determining the credibility of web health information- a Protocol of a gold standard approach. Front Digit Health 2021;3:801204. 35005698 10.3389/fdgth.2021.801204 PMC8732749

[62] Tsironis LK. Educational websites quality assessment framework. International Journal of Decision Sciences, Risk and Management 2021;10(1/2):51–77.

[63] Stuij SM, Drossaert CHC, Labrie NHM, et al.; INSTRUCT project group. Developing a digital training tool to support oncologists in the skill of information-provision: A user centred approach. BMC Med Educ 2020;20(1):135–117. 32357886 10.1186/s12909-020-1985-0 PMC7195777

[64] van Swol LM, Chang C-T. Plain language formatting of health advice messages may help to increase accessibility and understanding through more elaboration and processing of the message. JAMA Pediatr 2023;177(9):892–893. 37548986 10.1001/jamapediatrics.2023.2698

[65] Watermeyer J, Thwala Z, Beukes J. Medical terminology in intercultural health interactions. Health Commun 2021;36(9):1115–1124. 32202159 10.1080/10410236.2020.1735700

[66] Suarez-Almazor ME, Belseck E, Homik J, et al. Identifying clinical trials in the medical literature with electronic databases: MEDLINE alone is not enough. Control Clin Trials 2000;21(5):476–487. 11018564 10.1016/s0197-2456(00)00067-2

